# High-responsivity graphene photodetectors integrated on silicon microring resonators

**DOI:** 10.1038/s41467-021-23436-x

**Published:** 2021-06-18

**Authors:** S. Schuler, J. E. Muench, A. Ruocco, O. Balci, D. van Thourhout, V. Sorianello, M. Romagnoli, K. Watanabe, T. Taniguchi, I. Goykhman, A. C. Ferrari, T. Mueller

**Affiliations:** 1 Vienna University of Technology, Institute of Photonics, Vienna, Austria; 2 Cambridge Graphene Centre, University of Cambridge, Cambridge, UK; 3 Ghent University-IMEC, Photonics Research Group, Gent, Belgium; 4 Consorzio Nazionale per le Telecomunicazioni and Inphotec, Pisa, Italy; 5 National Institute for Materials Science, Tsukuba, Japan; 6 Technion—Israel Institute of Technology, Haifa, Israel

**Keywords:** Optical properties and devices, Integrated optics

## Abstract

Graphene integrated photonics provides several advantages over conventional Si photonics. Single layer graphene (SLG) enables fast, broadband, and energy-efficient electro-optic modulators, optical switches and photodetectors (GPDs), and is compatible with any optical waveguide. The last major barrier to SLG-based optical receivers lies in the current GPDs’ low responsivity when compared to conventional PDs. Here we overcome this by integrating a photo-thermoelectric GPD with a Si microring resonator. Under critical coupling, we achieve >90% light absorption in a ~6 *μ*m SLG channel along a Si waveguide. Cavity-enhanced light-matter interactions cause carriers in SLG to reach ~400 K for an input power ~0.6 mW, resulting in a voltage responsivity ~90 V/W, with a receiver sensitivity enabling our GPDs to operate at a 10^−9^ bit-error rate, on par with mature semiconductor technology, but with a natural generation of a voltage, rather than a current, thus removing the need for transimpedance amplification, with a reduction of energy-per-bit, cost, and foot-print.

## Abstract (web summary)

Optical receivers based on graphene still suffer from low responsivity. Here, the authors integrate a photo-thermoelectric graphene photodetector with a Si micro-ring resonator, and obtain a voltage responsivity ~ 90 V/W and a reduction of energy-per-bit consumption, enabling performance on par with mature semiconductor technology.

## Introduction

The same-chip integration^1^ of active and passive optical components with electronics offers a cost- and energy-efficient solution for short- and long-reach optical interconnects^2,3^. Single layer graphene (SLG) is an ideal material for integrated photonics^4,5^, promising e.g. high-speed (>200 GHz)^6,7^ and broadband (ultraviolet to far-infrared)^8,9^ operation that could lift bandwidth (BW) (~100 GHz)^10,11^ and spectral (<1600 nm)^12^ limitations of existing technologies, such as Ge/Si^13,14^ and InGaAsP/InP^15,16^. A variety of waveguide (WG)-integrated SLG-based photonic devices have been reported^17–34^, including electro-absorption (EAMs)^17–19^ and electro-refraction modulators (ERMs)^20^, optical switches^4,21^, and photodetectors (GPDs)^22–33^. SLG and layered materials can be integrated with passive Si photonic WGs^22–27^ or any other passive WG technology^4^, including Si_3_N_4_^29,34,35^, sapphire^36^, Ge^37^, and polymers^38,39^, extending the spectral range and scope of possible applications^37,40^.

SLG’s optical absorption is ~2.3% under normal incidence^41^, which limits the photoresponse in top-illuminated GPDs^8^. This can be increased in a WG configuration through the interaction with the evanescent field of the optical WG mode^42^. However, since the mode-overlap with SLG’s monatomic cross-section typically restricts absorption to ~0.01–0.1 dB/μm (~0.2–2%/μm)^4^, device lengths ~100 μm are needed for near-complete (>90%) absorption, with adverse effects on foot-print and capacitance^7^. The resulting trade-off between length and absorption has implications for GPDs that operate via the photo-thermoelectric effect (PTE)^43–45^: Due to slow (~ps^46^) heat dissipation to the lattice via phonon mediated cooling^46,47^, photo-excitation leads to the formation of a hot-carrier distribution in SLG^8,43,44^. The associated electron temperature, *T*_*e*_, can be substantially above the lattice temperature, *T*_0_^48^, and leads to a photovoltage^43–45^:

1 $${V}_{{\rm{PTE}}}=\int S(x)\cdot \nabla {T}_{e}(x){\rm{d}}x$$

if both a *T*_*e*_ gradient and a spatially varying Seebeck coefficient, *S*, (controlled by the chemical potential, *μ*_*c*_) are present^43^. In order to achieve a high (>mV) *V*_PTE_, it is better to absorb the incident electromagnetic energy over small (<10 *μ*m) lengths, leading to localised electronic heating for a higher (~tens K) *T*_*e*_ and ∇ *T*_*e*_(*x*).

Different approaches have been explored to increase and confine light absorption in free-space-coupled^49–51^ and WG-integrated GPDs^26–29^, e.g. by embedding SLG into optical cavities^50,51^, slot WGs^26^, plasmonic structures^28,29,31,49^, or by enhancing the light-matter interaction using sub-wavelength structures^27^, but these have coupling and propagation losses^31^, limitations in field enhancement^25,30^, or in carrier mobility *μ*^26,32^, fabrication flows incompatible with complementary metal-oxide-semiconductor (CMOS) processing^25,31,32^, bias-induced dark currents^30,31^, or a combination thereof^25,31,32^. Thus, the demonstration of GPDs on photonic integrated circuits (PICs) that leverage SLG’s unique hot-carrier dynamics and maximise the voltage responsivity *R*_[V/W]_ = *V*_PTE_/*P*_in_, with *P*_in_ the incident optical power in the WG, is challenging.

Here, we report GPDs integrated on looped WGs, known as microring resonators^52^, which act as PIC-embedded resonant cavities. The conversion of incident light into an electrical signal occurs via the PTE effect. The GPDs directly generate a voltage, which allows us to operate them without bias and dark currents, limiting the GPD noise to thermal Johnson-noise, due to fluctuations of the carrier density^53^. Other sources of noise, in particular low (~Hz-kHz) frequency contributions, such as 1/f and generation-recombination^54^, are not relevant for bias-free high-speed PTE-GPDs operating in the GHz regime^33^. This removes the need of transimpedance amplifiers (TIA) in the read-out electronics, with a reduction of the energy-per-bit cost and system foot-print. With *R*_[V/W]_ ~ 90 V/W, our GPDs pave the way towards SLG integration on Si photonic receivers, overcoming the limitation of photocurrent (*I*_ph_) generating GPDs with current responsivities *R*_[A/W]_ = *I*_ph_/*P*_in_ lower than mature Ge PDs^25,30,31^. We attribute this to our high (>10^4 ^cm^2^/Vs) *μ*, and the combination with the Si microring resonator giving a ~10-fold enhancement of electric field strength and >90% light absorption in just ~6 μm SLG on Si WGs.

## Results

### Device concept

**Fig. 1:**
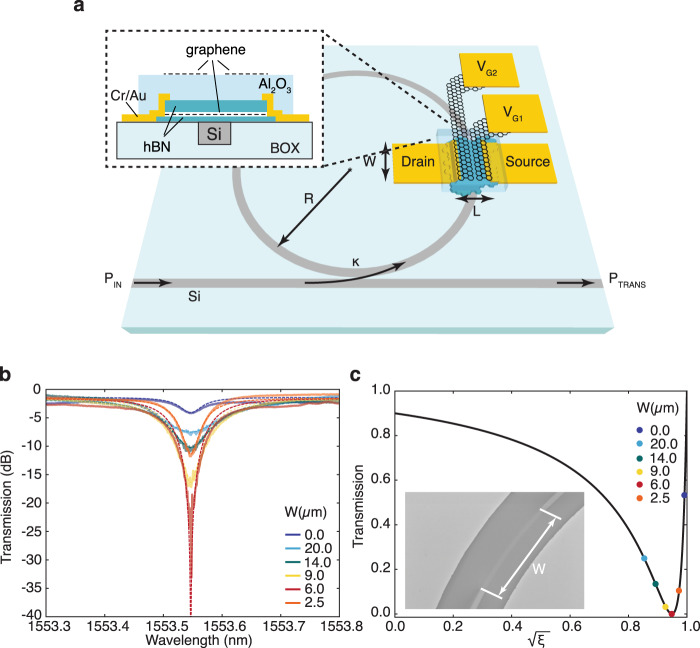
Si ring resonator integrated GPD. **a** Sketch of device. **b** Transmission spectra of a ring cavity with SLG on top, indicating the effects of varying *W* on ER and Q. For clarity, the spectra are aligned at the resonance wavelength closest to 1553.55 nm. **c** Corresponding transmission at resonance. The black line is the calculated transmission for *κ* = 10%. The coloured dots mark the transmission for various loss coefficients.

Figure 1 a is a scheme of our GPDs, which comprise a layered materials heterostructure (LHM) of SLG and hexagonal boron nitride (hBN). To increase the generated *V*_PTE_ upon optical illumination according to Eq. (1), encapsulation of the SLG channel in hBN ensures a high (>10^4 ^cm^2^/Vs) *μ*^55^ for large (~200 *μ*V/K) peak *S*^29^, according to Mott’s formula^43,44^:

2 $$S=-\frac{{\pi }^{2}{k}_{{\rm{B}}}^{2}{T}_{e}}{3e\sigma (\mu ,{\mu }_{c})}\frac{{\rm{d}}\sigma (\mu ,{\mu }_{c})}{{\rm{d}}{\mu }_{c}}$$

where *k*_B_ is the Boltzmann constant, *e* the electron charge, *σ* = *nμe* the conductivity, and *n* the carrier concentration. Dual-gate SLG electrodes, separated from the LMH by an Al_2_O_3_ layer, are employed to tune *S* in adjacent regions of the device^26^. The LMH is contacted on opposite sides and centrally aligned to the WG of a microring resonator fabricated on a Si-on-insulator (SOI) wafer. The resonator serves a two-fold purpose. First, the higher (compared to the bus WG) intra-cavity energy density^52^ results in ~10-fold enhanced light-matter interaction^56^ and can enable near-complete light absorption in the SLG channel if its coverage of the resonator is optimised. Second, the wavelength, *λ*, selectivity of the resonator^56,57^, makes the GPD suitable for wavelength division multiplexing (WDM)^14^, whereby the data rate of a single optical channel is increased by combining signals of different *λ* at the transmitter, and separating them at the receiver^58–60^.

To find the SLG length, *W,* over the ring that enables maximum absorption inside the resonator, we perform an initial experiment on a reference microring cavity with identical parameters (WG thickness *t*_WG_ = 220 nm, WG width *w*_WG_ = 480 nm, ring radius *R* = 40 *μ*m). Coupling to the resonator occurs through a 200 nm gap via a single bus WG, Fig. 1a, which has grating couplers (GCs) on either end, with coupling efficiency^14 ^$\eta ={P}_{{\rm{in}}}/{P}_{{\rm{fibre}}} \sim$0.28, with ${P}_{{\rm{fibre}}}$ the optical power in the fibre connecting source and SOI chip, determined from transmission measurements on reference WGs on the same chip. The power coupling between these two structures depends on the coupling and transmission coefficients, i.e. the scattering matrix elements relating incoming and outgoing electric fields from the coupling region^56,57^. The choice of the power coupling coefficient, *κ*, affects both the optical (frequency) bandwidth *f*_FWHM_ and the quality factor Q = *f*_FWHM_/*f*_res_, with *f*_res_ the resonance frequency^59^ of the resonator, resulting in a trade-off between achievable extinction ratio (ER, defined as the ratio of minimum (at resonance) and maximum transmitted optical power^50^), thus *R*_[V/W]_, and the maximum achievable electrical bandwidth, *f*_3dB_. For our GPD, we select *κ* = 10% (with a measured optical (wavelength) bandwidth *λ*_FWHM_ ~ 150 pm at *λ*_res_ ~ 1.55 μm, corresponding to *f*_FWHM_ ~ 18.7 GHz. This, according to^61^:

3 $${f}_{{\rm{3dB}}}=\sqrt{\sqrt{2}-1}\times {f}_{{\rm{FWHM}}}$$

allows for *f*_3dB_ ~ 12 GHz in our design, sufficient for applications in data centre optical interconnects^2^.

The wavelength-dependent transmitted power, *P*_trans_, of a ring resonator can be written as^52^:

4 $${P}_{{\rm{trans}}}={P}_{{\rm{in}}}\frac{(1-\kappa )+\xi -2\sqrt{(1-\kappa )\xi }\cos (\theta )}{1+(1-\kappa )\xi -2\sqrt{(1-\kappa )\xi }\cos (\theta )}$$

where *θ* = 4*π*^2^*Rn*_eff_/*λ* is the round-trip phase shift of the circulating mode and the effective mode index *n*_eff_ = *β*_*m*_/*k*_0_, with *β*_*m*_ the propagation constant of the mode, defined as the wavevector component along the WG, and *k*_0_ the free-space wavevector^60,62^. Omitting negligible losses caused by coupling between bus and ring, the term:

5 $$\xi ={e}^{-W{\alpha }_{{\rm{SLG}}}}{e}^{-2\pi R{\alpha }_{{\rm{WG}}}}$$

describes the round-trip propagation loss in the ring, with *α*_SLG_ and *α*_WG_, in dB/*μ*m, the power attenuation coefficients in SLG and Si WG, respectively. When *θ* = 2*πm* (*m* = 1, 2, 3…), the light in the ring constructively interferes with itself and the cavity is in resonance^52^. From Eq. (4), the transmission drops to zero if *ξ* = 1 − *κ*. Under this so-called critical coupling^57^, as the transmission approaches zero, maximum absorption inside the ring resonator is achieved. With all other parameters fixed in Eq. (5), changing the SLG-induced losses by changing *W* can therefore be used to tune *ξ* and achieve critical coupling.

### Coupling and absorption optimisation

To find the optimum *W*, we first measure the transmission of an unloaded (no SLG, i.e. *W* = 0 *μ*m) resonator by coupling light (continuous-wave (CW), TE-polarised) from a tunable laser (Newport TLB6700) into the bus WG, using an optical single-mode fibre, and measuring the transmitted power at the output GC as a function of *λ*, around one of the resonance peaks close to main Telecom wavelength at 1.55 *μ*m, Fig. 1b. Due to SLG’s broadband absorption^8^, there will be identical behaviour for the other resonances, apart from a shift in *P*_in_ with GC response envelope^14^. The results, after calibration for the coupling losses, are shown by the dark-blue line and symbols in Figs. 1b, c, respectively. The microring resonator is not critically coupled at resonance for *λ* ~ 1553.55 *μ*m, as *P*_trans_ does not vanish, but only part of the incident power is dissipated in the WG. From Eqs. (4), (5), *α*_WG_ ~ 1.4 dB/cm.

We then proceed to study the effect of SLG with varying *W* on the power dissipated in the resonator. We first place a *W* = 20 *μ*m SLG flake, prepared by micro-mechanical cleavage (MC)^63^ of bulk graphite, transferred using a micro-manipulator and a stamp consisting of polycarbonate (PC) and polydimethylsiloxane (PDMS), and cleaned by immersion in chloroform, over the ring, and measure the transmission as before. Using successive electron beam lithography (EBL, Raith e-LINE) runs to define a poly(methyl methacrylate) (PMMA) etch mask and reactive ion etching in O_2_ to remove excess material, we then reduce *W* further in several steps down to 2.5 *μ*m, with transmission measurements in between. The results, Figs. 1b, c, show an initial transmission decrease at resonance with decreasing *W*, before the trend is inverted as *W* tends to zero. The minimum transmission, indicating critical coupling, is for *W* = 6 *μ*m. From Eq. (5) we extract *α*_G_ ~ 0.07 dB/*μ*m, in agreement with measured^17^ and simulated^4^ values from literature. Using these and the comparison of the transmission curves for *W* = 6 *μ*m, we estimate the fraction of absorbed light in the SLG channel to be ~92% under critical coupling. Figure 1b can also be used to monitor the effects of changing *W* on *Q*, as discussed in Supplementary Note 1. Supplementary Fig. 1 plots the degradation of *Q* as losses are increased with *W*. We further use *Q*^56,64^ to confirm the absorption in our devices.

### LMH characterisation

**Fig. 2:**
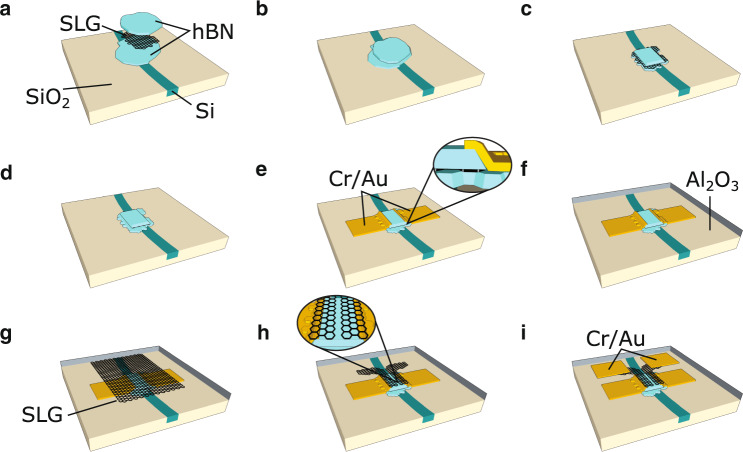
Device fabrication. **a** Assembly of hBN/SLG/hBN. **b** Stack placement on photonic circuit and interface cleaning. **c** hBN etching in SF_6_ plasma. **d** SLG etching in O_2_ plasma to define channel geometry. **e** Metallization (Cr/Au) for drain-source contacts. **f** Al seed layer evaporation and ALD of Al_2_O_3_. **g** Wet transfer of CVD SLG. **h** Split-gate fabrication. **i** Metallization (Cr/Au) for gate contacts.

**Fig. 3:**
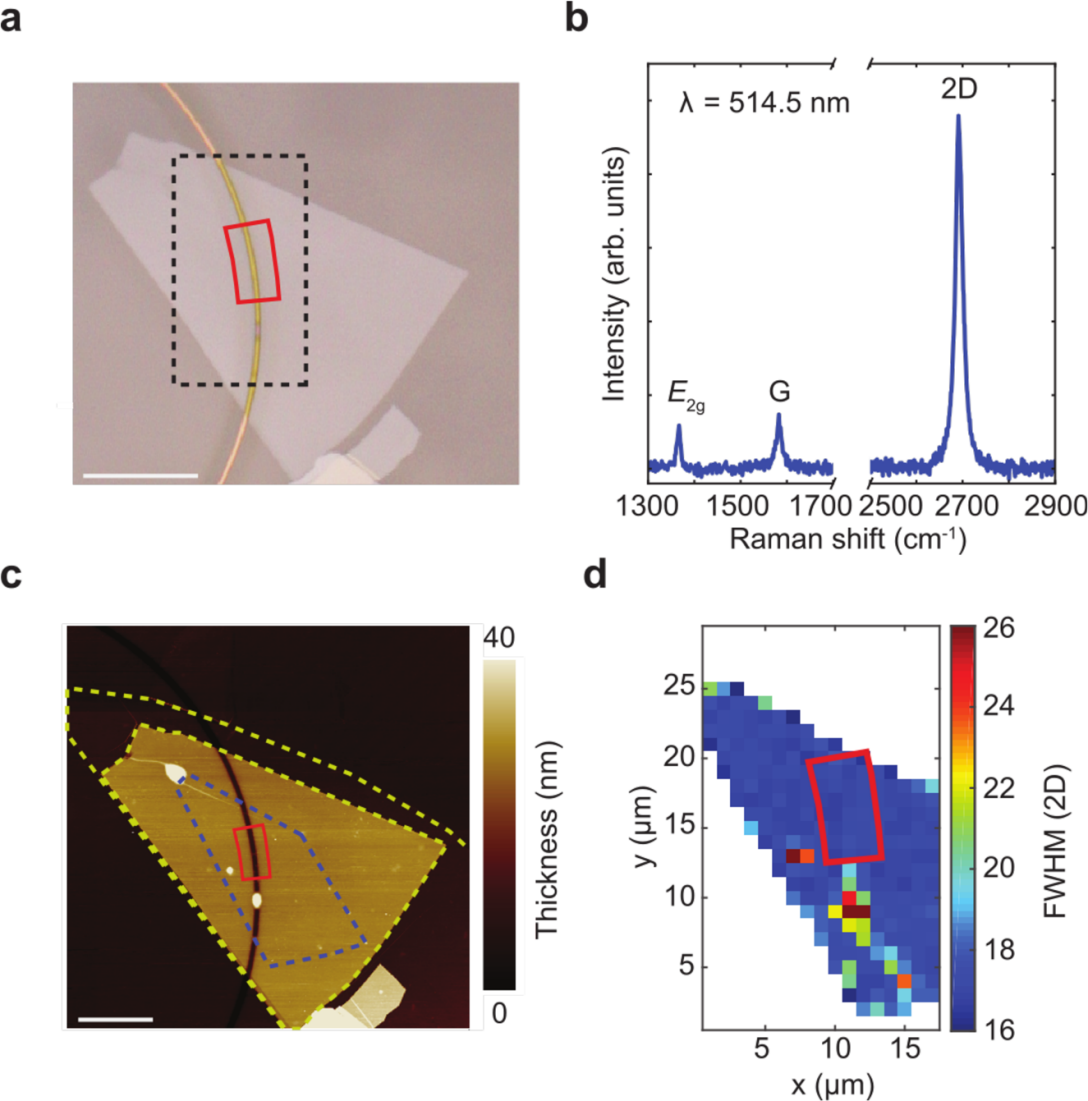
LMH characterisation. **a** Microscope image of hBN/SLG/hBN on ring resonator. The black dashed line indicates the area over which the Raman map in **d** is measured. Scale bar, 10 μm. **b** Raman spectrum measured at the position of the final device. **c** AFM image of LMH. The yellow dashed line indicates the area of top and bottom hBN. The blue line indicates the SLG area. Scale bar, 10 μm. **d** Raman map of FWHM(2D). The red box marks the position of the final device in **a, c, d**.

Based on these findings, we fabricate the GPD in Fig. 1a with *W* = 6 *μ*m from a LMH (hBN encapsulated SLG as channel layer) on top of the ring resonator, as shown in Fig. 2 and described in Methods. A microscope image of hBN/SLG/hBN on the WG is in Fig. 3a. We perform Raman spectroscopy (Renishaw inVia at 514.5 nm, power <0.5 mW) and atomic force microscopy (AFM, Bruker Dimension Icon) to monitor the SLG quality. A typical Raman spectrum before further processing of the stack is in Fig. 3b. The position of the combined hBN E_2g_ peaks^65^ from top and bottom flakes is Pos(*E*_2g_) ~ 1366 cm^−1^ with full-width half maximum, FWHM(*E*_2g_) ~ 9.5 cm^−1^, as expected considering the top flake is bulk and that the planar domain size in MC-produced hBN crystals is limited by the flake size^55,66^. Pos(2D) ~ 2693 cm^−1^, FWHM(2D) ~ 18 cm^−1^, Pos(G) ~ 1583 cm^−1^, FWHM(G) ~ 14 cm^−1^, confirming the presence of SLG and low *n* < 10^12^ cm^−2^^67^. The area (A(2D)/A(G) ~ 10.7) and intensity (I(2D)/I(G) ~ 7.6) ratios indicate a Fermi level *E*_F_ < 100 meV^67–69^.

The AFM scan of the overlap region between LMH and microring in Fig. 3c shows blister-free SLG/hBN interfaces, confirming successful cleaning^55^, apart from a bubble trapped in a cladding trench above the WG. The FWHM(2D) map in Fig. 3d, taken from a 20 × 30 *μ*m^2^ area in the centre of the LMH, shows a region with homogeneous (spread < 1 cm^−1^) and narrow (≤18 cm^−1^) FWHM(2D) and spots of increased (>21 cm^−1^) FWHM(2D) that coincide with the blister position, as revealed by AFM. Based on these findings, we then select the channel position (marked red in Figs. 3a, c, d) to be in a blister-free region. The final device has an active area *L* × *W* ~ 2.5 × 6 *μ*m^2^. We use *L* ~ 2.5 *μ*m, of the order of twice the cooling length *L*_cooling_ in SLG (~1 *μ*m^25,70^, related to electron thermal conductivity *κ*_*e*_ (see Methods) and interfacial heat conductivity Γ ~ 0.5–5 MWm^−2^K^−1^ ^71,72^, via ${L}_{{\rm{cooling}}}=\sqrt{{\kappa }_{e}/{{\Gamma }}}$^44^), to fully exploit the *T*_*e*_ profile with expected maximum at *L*/2^26,29^, i.e. the WG centre.

### Electrical characterisation

**Fig. 4:**
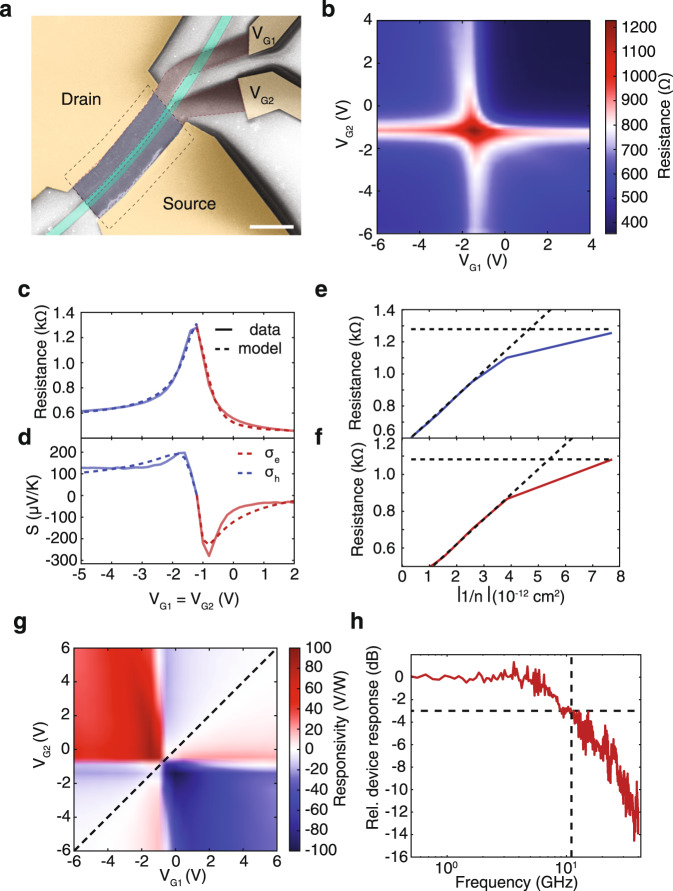
GPD characterisation. **a** False-colour SEM image of our GPD, showing Cr/Au contacts (yellow), Si WG (green), contacted hBN/SLG/hBN (blue, dashed line under contacts), and SLG gates (red). Scale bar: 2 μm. **b** Resistance map demonstrating independent tunability of charge carrier concentration in the SLG channel via *V*_G1_ and *V*_G2_. **c** Electrical characterization at homogeneous channel doping (solid lines, measured data; dashed lines, model). **d** Calculated *S* based on the electrical data in **c**. **e**, **f**) Resistance vs. inverse carrier concentration for **e** electron and **f** hole doping. **g** Photoresponse at zero bias on resonance (*λ* = 1555.87 nm). **h** Frequency response. The 3-dB cutoff frequency, marked by intersecting dashed lines, is ~12 GHz.

An image of the full GPD after fabrication, obtained by SEM, is in Fig. 4a, revealing a well-aligned WG, LMH, and split-gate structure. We first verify gate tunability of the SLG channel by measuring the drain-source current (*I*_DS_) at a fixed drain-source voltage (*V*_DS_) while varying the two gate-voltages. The resulting resistance map (Fig. 4b) shows a cross pattern, which confirms that four junction constellations (p-n, n-p, n-n, p-p) can be generated in the channel^26^. In order to extract the contact resistance, *R*_contact_, and *μ* from the GPD directly (rather than from a four-probe reference structure made from a second LMH), then used to estimate *S*, we utilize the measured transfer curve at homogenous channel doping in Fig. 4c and plot the device resistance as function of the inverse carrier concentration (1/*n*) for electron (Fig. 4e) and hole (Fig. 4f) doping. By fitting the linear part (as 1/*n* → 0) of these plots, as for ref. ^73^, *R*_contact_ can be obtained from the intersection of the fit curve and y-axis, while the residual carrier concentration, *n*_0_, is found from the intersection between the fit curve and a horizontal line through the maximum. Using these values, we then model the total device resistance as for ref. ^26^: *R*_total_ = *R*_contact_ + $\frac{L}{W}\frac{1}{e\mu n}$, with $n=\sqrt{{n}_{0}^{2}+{\left[{C}_{{\rm{ox}}}/e\left({V}_{{\rm{G}}}-{V}_{{\rm{CNP}}}\right)\right]}^{2}}$, where *R*_contact_ includes the contacts and the contribution from the ungated region, *V*_CNP_ is the gate voltage corresponding to the charge neutrality point (CNP, *E*_F_ = 0 meV), *C*_ox_ is the gate capacitance, and *μ* is used as a fit parameter. The original data (solid line) and the model (dashed line) are compared in Fig. 4c. We get *R*_contact_ ~ 400 Ω and ~530 Ω, as well as *μ*_e_ ~ 17, 700 cm^2^/Vs and *μ*_h_ ~ 11, 800 cm^2^/Vs for electrons (red lines) and holes (blue lines), respectively. This demonstrates a PIC-integrated, LMH-based PD with high (>10^4^ cm^2^/Vs) *μ*, while refs. ^25,74^ used hBN encapsulation for alternative detection concepts.

### Steady-state photoresponse

For optical characterisation, we first couple modulated light (ON-OFF) with a duty cycle of 50% from a tuneable laser source (Agilent 81680A) into the bus WG using an optical single-mode fibre. While varying the potential at the two gate electrodes (*V*_G1_, *V*_G2_), the photoresponse of the unbiased GPD (*V*_DS_ = 0 mV, to avoid dark currents impairing the noise performance) is recorded using a lock-in amplifier. Since the PTE effect as electromotive force is intrinsically best read-out as open circuit potential difference^4,75^, we record the response as *V*_PTE_, rather than measuring the resulting short-circuit photocurrent, which depends on external factors, such as *R*_contact_. Figure 4g shows a photoresponsivity map measured on resonance at 1555.87 nm, from which we extract a maximum *R*_[V/W]_ ~ 90 V/W. The six-fold pattern, with the highest photoresponse for bipolar (p-n, n-p) junctions, and a sign-change across the diagonal (*V*_G1_ = *V*_G2_) for unipolar (n-n, p-p) junctions in the SLG channel, confirms that the PTE effect dominates the conversion of photons into electrical signal (rather than a photovoltaic conversion with a two-fold pattern in photovoltage over the same measurement range)^43,44^. Our *R*_[V/W]_ outperforms the current state-of-the-art for waveguide-integrated PTE-GPDs, *R*_[V/W]_ ~ 3–12 V/W^25,29,33,76^, by around one order of magnitude.

### High-speed photoresponse

To determine the BW, we modulate CW light at 1555.87 nm from the same source using a commercial (Thorlabs LN05S-FC) intensity modulator (lithium niobate, *f*_3dB_ = 40 GHz) and couple it into the device. While tuning the modulation frequency of the external modulator, we monitor the GPD response with an electrical spectrum analyzer (Agilent PSX N9030A), while the gate bias (*V*_G1_ = −0.5 V, *V*_G2_ = −2.1 V) is set at an operating point where *R*_[V/W]_ is largest. This gives a 3-dB bandwidth ~12 GHz, Fig. 4h, as expected from the design of the passive photonic structure and the cavity-imposed limit calculated via Eq. (3).

### Power and wavelength dependence

**Fig. 5:**
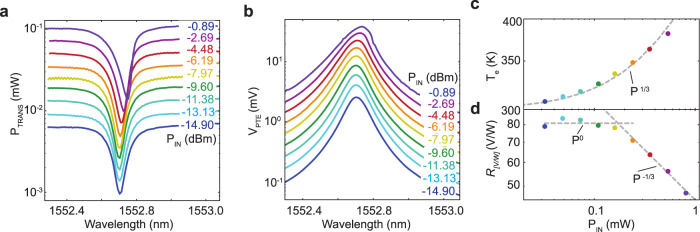
Wavelength and power dependence. **a** Transmitted power for various *P*_in_. **b** Photovoltage for a fixed gate voltage combination (*V*_G1_ = 1 V, *V*_G2_ = − 1 V) for various input powers (same color code as for the transmission in **a**). **c** *T*_*e*_ calculated from the photoresponse in **b** and *S* in Fig. 4d. **d** Power-dependent *R*_[V/W]_.

Figures 5a, b plot the wavelength dependence of optical transmission and photovoltage for *V*_G1_ = 1 V, *V*_G2_ = −1 V and various *P*_in_. The maxima of the *V*_PTE_ traces match the resonance minima in *P*_trans_, confirming the proportionality between the two, as SLG dominates absorption for our resonator-based PDs. This is also shown in Supplementary Note 2 and Supplementary Fig. 2, where we plot the estimated absorption and resulting *V*_PTE_ at low (<0.1 mW) *P*_in_ on a linear scale. The shift of the resonance and its asymmetry for higher *P*_in_ (>0.2 mW) are attributed to the power-dependent change of the effective refractive index of the Si WG through a thermo-optic effect^52,77^. Extracting the power-dependent maxima in *V*_PTE_ allows us to estimate *T*_*e*_ at resonance, and plot the *R*_[V/W]_ power dependence.

The junction carrier temperature, *T*_e,j_, can be written as (see Methods):

6 $${T}_{{\rm{e,j}}}=\sqrt{2\left|\frac{{V}_{{\rm{PTE}}}}{{\zeta }_{1}-{\zeta }_{2}}\right|+{T}_{0}^{2}}$$

where *T*_0_ = 294 K is the room temperature and ${\zeta }_{1,2}=\frac{{\pi }^{2}{k}_{{\rm{B}}}^{2}}{3e\sigma }\frac{{\rm{d}}\sigma }{{\rm{d}}\epsilon }$ at *ϵ* = *E*_*F*_, in analogy to Eq. (2). In order to estimate *ζ*_1,2_, following the same method used to determine *S* in Fig. 4d, we use *R*_contact_ and *μ* as obtained from the electrical measurements at homogeneous channel doping. The resulting *T*_*e*_ extracted for different *P*_in_ is in Fig. 5c. The carriers reach *T*_*e*_ ~ 400 K for *P*_in_ ~ 0.6 mW, due to cavity-enhanced light-matter interaction. The *T*_*e*_ power dependence can be fitted by the heat equation^44,70^, neglecting diffusive cooling through the contacts (see Methods):

7 $${T}_{{\rm{e}},{\rm{j}}}={(\beta {P}_{{\rm{IN}}}+{T}_{0}^{\delta })}^{\frac{1}{\delta }}$$

with *δ* ~ 3^78^ and *β* a fitting parameter.

The associated, power-dependent, *R*_[V/W]_ is in Fig. 5d. For small *P*_in_ (<0.2 mW), we get a constant *R*_[V/W]_. For *P*_in_ > 0.2 mW we have sublinear scaling between *V*_PTE_ and *P*_in_, giving a *R*_[V/W]_ drop according to ${R}_{[{\rm{V}}/{\rm{W}}]}\propto {P}_{{\rm{in}}}^{-1/3}$ due to the *T*_*e*_ dependence of the electronic heat capacity^78,79^.

## Discussion

In order to assess our GPD performance against non-SLG-based ones, where typically a photocurrent *I*_ph_ is generated^14^, we compare two representative optical receiver implementations: a conventional (i.e. non-SLG) system based on a wafer-scale, commercial, high-speed (>50 GHz) Ge photodiode with *R*_[A/W]_ ~ 0.5 A/W^80^, and a receiver based on our GPD. In both systems the same amplifier is employed to obtain a >200 mV output voltage swing (*V*_OUT_), as required for driving the subsequent clock and data recovery CMOS circuit. For simplicity we assume that the amplifier represents a capacitive load.

Our GPDs are based on the PTE effect, where an electromotive force directly provides a voltage, rather than a current^8,79^. In case of Ge, an additional TIA is needed to convert the photocurrent into a voltage for further signal processing^81^. In the TIA we consider a feedback resistor *R*_F_ = (90 V/W)/(0.5 A/W) = 180 Ω, which assures the same *V*_OUT_ for same optical input power in both cases. Neglecting any noise other than thermal noise produced by *R*_F_, we estimate for the conventional receiver a lower limit for the sensitivity ${\bar{P}}_{{\rm{sens}}}=Q{i}_{{\rm{n}}}/{R}_{{\rm{[A/W]}}}=12.6\,\mu$W ~ −19 dBm at a bit-error-rate (i.e. probability of false identification of a bit by the receiver decision circuit^82^) BER = 10^−9^. We calculate the thermal noise current as *i*_n_ = $\sqrt{4{k}_{{\rm{B}}}\cdot T\cdot BW/{R}_{{\rm{F}}}}$ with BW = 12 GHz, as in our GPDs, and a *Q* factor (i.e. required signal-to-noise ratio to get a specific BER^83^) ~6 from^82^ BER = $\frac{1}{2}{\rm{erfc}}(Q/\sqrt{2})$. For our GPDs, we estimate ${\bar{P}}_{{\rm{sens}}}$=*Qv*_n_/*R*_[V/W]_ ~ −16 dBm for same BER and BW, where *v*_n_=$\sqrt{4{k}_{{\rm{B}}}\cdot T\cdot BW\cdot {R}_{{\rm{G}}}}$, and *R*_G_ ~ 800 Ω is the total device resistance. Thus, in contrast to previous reports of both PTE^25–27,29,33,76^ and non-PTE^30–32^ GPDs for integrated photonics, ${\bar{P}}_{{\rm{sens}}}$ of our GPD-based receiver is on par with mature semiconductor technology, and could be further improved by reducing *R*_contact_, which dominates the total device resistance, thus being the primary source of thermal noise. The natural generation of a voltage makes the need for a TIA obsolete, with a reduction of energy-per-bit cost and system foot-print.

In summary, we reported photo-thermoelectric GPDs, integrated on Si microring resonators. By tuning the SLG coverage on top of the resonator, we optimised the round-trip propagation losses inside the cavity to get critical coupling, achieving >90% light absorption in ~6 *μ*m SLG. The resulting carrier heating on such compact lengths enables high peak *T*_*e*_ ~ 400 K in the GPDs. In combination with high (>10^4^ cm^2^/Vs) mobility, obtained by encapsulating the SLG channel in hBN, this allowed us to get *R*_[V/W]_ ~ 90 V/W. Our bias-free, Johnson-noise limited GPDs, with voltage output, are a more power-efficient alternative to state of the art commercial PDs used in optical interconnects. Hot-carrier effects in SLG can be exploited for receiver architectures where current-to-voltage conversion, traditionally performed by transimpedance amplifiers, can be bypassed for a reduction in energy-per-bit cost and system foot-print.

## Methods

### Sample fabrication

The LMH is made as follows^55^: SLG and hBN flakes of different thicknesses (*t*_bottom_ ~ 3 nm, *t*_top_ ~ 20 nm) are prepared on Si/SiO_2_ (${t}_{{{\rm{SiO}}}_{2}}$ = 285 nm) by MC of bulk graphite (Graphenium) and hBN single crystals grown at high pressure and temperature as detailed in ref. ^84^. The thickness of the bottom hBN, *t*_bottom_, is chosen with the following trade-off: sufficiently thin (<5 nm) to ensure *α*_SLG_ (thus *ξ*) comparable to the initial experiment used to find *W*, but sufficiently thick (~nm) that high (>10^4^ cm^2^/Vs) *μ* is achieved, due to reduced (in comparison to SiO_2_) carrier inhomogeneities, roughness, and charge impurities^55,85,86^. A micro-manipulator and a PC/PDMS stamp are then used to pick up and stack the flakes at 50 °C (Fig. 2a). In order to clean the LMH interfaces, the target photonic chip is then heated to 180 °C^55^, while we align the LMH to the Si WG. We then laminate the PC film onto the target substrate, pushing contamination blisters, formed at the SLG/hBN interfaces, out of the GPD channel region and placing the LMH on the WG, Fig. 2b.

After the PC film is dissolved in chloroform, we perform EBL (Raith EBPG 5200) to define the GPD channel geometry via a PMMA etch mask. To transfer this pattern to the LMH, we use two dry etching steps. First, to achieve etch selectivity between LMH and underlying photonic circuit, we use a reactive ion etcher (RIE, Plasma-Therm) with a forward radio frequency power ~80 W and an SF_6_ flow ~80 sccm. As reported by ref. ^87^, these conditions allow fast (>200 nm/min) etching of hBN, slow (<7 nm/min) etching of SiO_2_, while SLG is not etched, serving as etch stop on the bottom hBN flake (Fig. 2c). We then expose the LMH to low power (3W) O_2_ plasma to remove all excess SLG, leaving behind the fully shaped GPD channel (Fig. 2d). A second EBL step, electron beam evaporation (5nm Cr/50nm Au), and lift-off in acetone are then used to contact the exposed SLG channel edges (Fig. 2e). To fabricate the split-gate structure on top of the LMH, required to create a p-n juntion in the GPD channel, a transparent (at *λ* ~ 1.55 *μ*m) conductor that does not affect *ξ* (thus *W*) is needed. A second layer of SLG, sufficiently high (~tens nm) above the WG to leave *α*_SLG_ unaltered, can be used for this. We therefore thermally evaporate 1nm Al as seed layer on the top hBN, and atomic layer deposit (ALD, Savannah) *t*_ox_ ~ 20 nm Al_2_O_3_ as additional gate dielectric and spacer between channel and gate electrodes (Fig. 2f). The combined thickness of the top-gate dielectric (*t*_top_ + *t*_ox_) ensures both low (~2 V or less) gate biases at the operating point of the GPD and, from eigenmode *Lumerical* simulations, a change in *α*_SLG_ < 25%.

To ensure alignment between LMH and gate SLG, we transfer a continuous film of SLG, grown by chemical vapor deposition (CVD) on Cu, following the process described in ref. ^88^, using a PMMA support membrane^89^ on the SLG/Cu substrate. We etch Cu in ammonium persulfate, transfer the PMMA/SLG stack onto the photonic chip (Fig. 2g), and remove the PMMA by immersion in acetone. We then use two additional EBL steps, O_2_ plasma etching, and electron beam evaporation, to define the SLG split-gate geometry (Fig. 2h) and fabricate metal contacts to these gates (Fig. 2i). Finally, we perform optical lithography on a laser writer (MicroTECH LW405) and wet etching in HF to get access to drain and source contact pads.

### Temperature estimation

The junction *T*_*e*_ can be extracted from power-dependent photovoltage measurements. Rewriting Eq. (2) as *S* = *ζT*_e_, where $\zeta =-({\pi }^{2}{k}_{{\rm{B}}}^{2})/3e\sigma {\rm{d}}\sigma /{\rm{d}}\epsilon$ at *ϵ* = *E*_*F*_, and using this in Eq. (1), we get *V*_PTE_ = ∫*S*(*x*) ∇ *T*_e_(*x*)d*x* = ∫*ζ*(*x*)*T*_e_(*x*) ∇ *T*_e_(*x*)d*x*. Integration over the p-n-junction gives ${V}_{{\rm{PTE}}}={\zeta }_{1}\mathop{\int}\nolimits_{-\frac{L}{2}}^{0}{T}_{{\rm{e}}}(x)\frac{{\rm{d}}{T}_{{\rm{e}}}}{{\rm{d}}x}{\rm{d}}x+{\zeta }_{2}\mathop{\int}\nolimits_{0}^{\frac{L}{2}}{T}_{{\rm{e}}}(x)\frac{{\rm{d}}{T}_{{\rm{e}}}}{{\rm{d}}x}{\rm{d}}x=\frac{{\zeta }_{1}-{\zeta }_{2}}{2}\left({T}_{{\rm{e,j}}}^{2}-{T}_{0}^{2}\right)$. From this, we extract the junction *T*_*e*_ from the measured photovoltage as *T*_e,j_=$\sqrt{2\left|\frac{{V}_{{\rm{PTE}}}}{{\zeta }_{1}-{\zeta }_{2}}\right|+{T}_{0}^{2}}$. In the most general case, the *T*_*e*_ profile can be calculated from the heat equation^44,70^, including diffusive cooling through contacts and cooling through the phonon bath: $\frac{{\rm{d}}q}{{\rm{d}}x}=-\frac{{\rm{d}}}{{\rm{d}}x}\left({\kappa }_{{\rm{e}}}\frac{{\rm{d}}}{{\rm{d}}x}{T}_{{\rm{e}}}\right)+\frac{{\kappa }_{{\rm{e}}}}{{L}_{{\rm{cooling}}}^{2}}\left({T}_{{\rm{e}}}^{\delta }-{T}_{{\rm{0}}}^{\delta }\right)$, where $\frac{{\rm{d}}q}{{\rm{d}}x}$ describes the heating of the system. The electronic thermal conductivity is given by ${\kappa }_{{\rm{e}}}=\sigma {{\mathcal{L}}}_{0}{T}_{{\rm{e}}}$^90^, with Lorenz number ${{\mathcal{L}}}_{0}$, while a ${T}_{e}^{\delta }$ dependence with *δ* ~ 3 is characteristic for SLG^70,91^. Assuming the distance from the heat source to the heat sink larger than the cooling length (*L*_channel_ > 2*L*_cooling_), the heat equation becomes $\frac{{\rm{d}}q}{{\rm{d}}x}=\frac{{\kappa }_{{\rm{e}}}}{{L}_{{\rm{cooling}}}^{2}}\left({T}_{{\rm{e}}}^{\delta }-{T}_{{\rm{0}}}^{\delta }\right)$, with $\frac{{\rm{d}}q}{{\rm{d}}x}=\alpha {P}_{{\rm{in}}}/A$, *αP*_in_ the fraction of the absorbed power, and *A* the heated area. We thus get ${T}_{{\rm{e,j}}}={\left(\beta {P}_{{\rm{in}}}+{T}_{0}^{\delta }\right)}^{\frac{1}{\delta }}$, with *β* a fit parameter.

## Supplementary information

Supplementary Information

## Data Availability

Data supporting the findings of this study are available from the corresponding authors upon reasonable request.
