# Supplementary material for: High-responsivity graphene photodetectors integrated on silicon microring resonators
Source: Nat Commun. 2021 Jun 18;12:3733. doi: 10.1038/s41467-021-23436-x (PMC8213857; doi:10.1038/s41467-021-23436-x)
Supplement: Supplementary file 1 — Supplementary Information [file 41467_2021_23436_MOESM1_ESM.pdf]

Supplementary Information for: High-responsivity graphene photodetectors integrated on silicon microring resonators

S. Schuler[1,2]*, J. E. Muench[2]*, A. Ruocco[2], O. Balci[2], D. van Thourhout[3], V. Sorianello[4], M. Romagnoli[4], K. Watanabe[5], T. Taniguchi[5], I. Goykhman[2,6], A. C. Ferrari[2]†, Thomas Mueller[1]†

[1] Vienna University of Technology, Institute of Photonics, Gusshausstrasse 27-29, 1040 Vienna, Austria
[2] Cambridge Graphene Centre, University of Cambridge, Cambridge CB3 0FA, UK
[3] Ghent University-IMEC, Photonics Research Group, Technologiepark-Zwijnaarde 126, 9052 Gent, Belgium
[4] Consorzio Nazionale per le Telecomunicazioni and Inphotec, via Moruzzi 1, 56124 Pisa, Italy
[5] National Institute for Materials Science, 1-1 Namiki, Tsukuba, 305-0044 Japan
[6] Technion - Israel Institute of Technology, Haifa, 3200003, Israel
* These authors contributed equally
† email:acf26@eng.cam.ac.uk
† email:thomas.mueller@tuwien.ac.at

Supplementary Figures

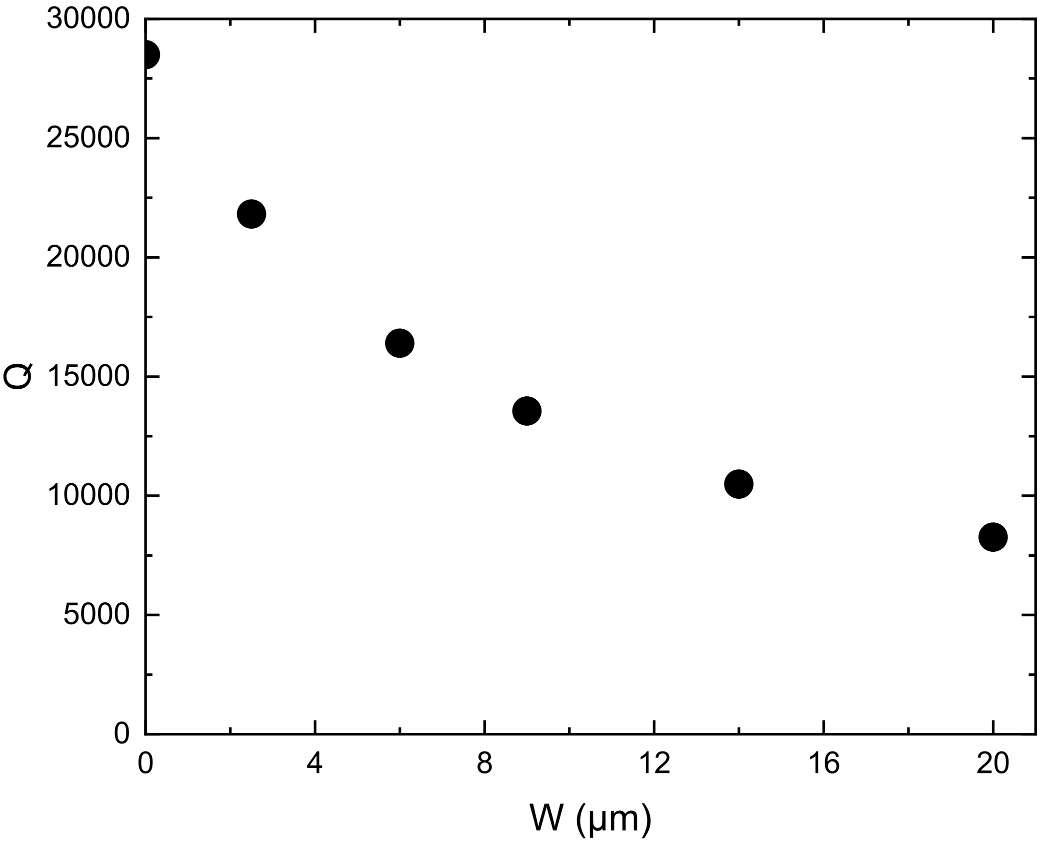

Supplementary Figure 1: Q, extracted from transmission spectra in Fig.1b, as function of W.

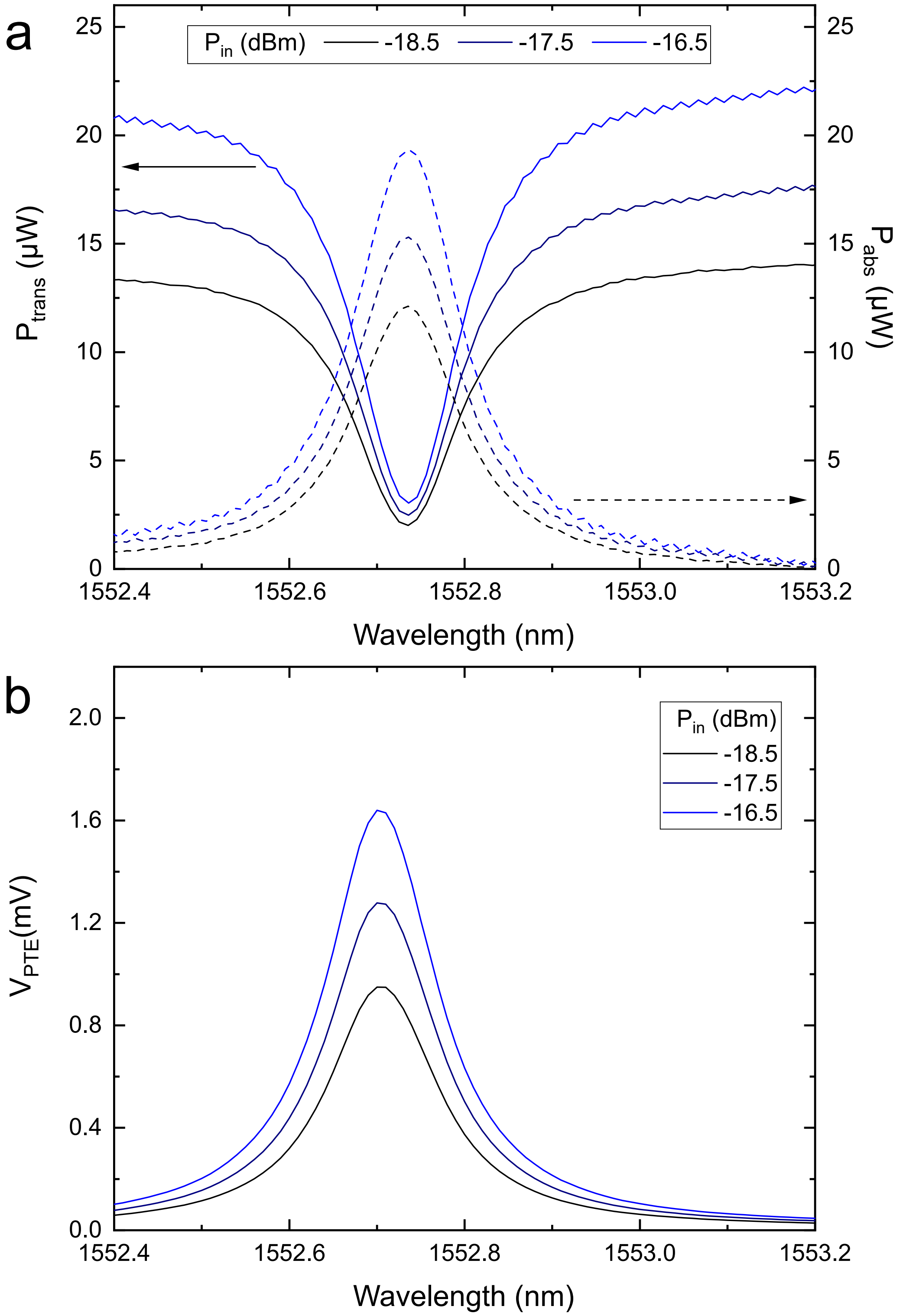

Supplementary Figure 2: (a) Transmitted (solid) and absorbed (dashed) power for three different P_{in}. (b) Corresponding photovoltage.

Supplementary Table

W (μm)	0	2.5	6	9	14	20
Q ($\times 10^4$)	2.8	2.2	1.6	1.4	1.0	0.8

Supplementary Table 1: Q for SLG-loaded MRR (cover length W).

Supplementary Note 1: Q from transmission spectra as a function of W

Supplementary Table 1 and Supplementary Fig.1 show Q extracted via[1]:

$$Q = \frac{\lambda_{\text{res}}}{\lambda_{\text{FWHM}}} \tag{1}$$

from Lorentzian fits of the transmission curves plotted in Fig.1b of the main text. The results confirm that the unloaded (W=0μm) MRR exhibits the highest Q, which decreases as W increases. E.g., for the maximum SLG loading in Fig.1b and Supplementary Fig.1 (W=20μm), Q is reduced$\sim$ 71% with respect to the maximum value. This degradation of Q gives a trade-off with the increase in absorption: as long as the MRR is under-coupled, absorption increases with W while Q decreases. Once critical coupling is achieved, and W is increased further, both Q and absorption decrease, as shown in Fig.1c and Supplementary Fig.1.

Supplementary Note 2: power absorbed in the GPDs

Supplementary Fig.2 shows a subset of the transmitted power (P_{trans}) and photovoltage (V_{PTE}) data of Fig.5a,b of the main text. Supplementary Fig.2a provides an estimation of the power absorbed in our GPD, extracted via the simplified expression $P_{\text{abs}} = P_{\text{in}} - P_{\text{trans}}$ [2]. The subset is restricted to three traces from the linear regime ($P_{\text{in}} < 0.1$mW) and plotted in linear scale, both chosen to allow a direct visual comparison between Supplementary Fig.2a and b. Supplementary Fig.2 shows the consistent proportionality between P_{abs} and V_{PTE} in our GPDs. The proportionality factor is given by the internal responsivity, defined as $R_{\text{int}} = V_{\text{PTE}}/P_{\text{abs}}$[3].

Supplementary References

[1] W. Bogaerts, P. De Heyn, T. Van Vaerenbergh, K. De Vos, S. Kumar Selvaraja, T. Claes, P. Dumon, P. Bienstman, D. Van Thourhout, and R. Baets, Laser & Photonics Reviews **6**, 47 (2012).

[2] D. G. Rabus, *Integrated ring resonators* (Springer, Berlin, 2007).

[3] F. H. L. Koppens, T. Mueller, P. Avouris, A. C. Ferrari, M. S. Vitiello, and M. Polini, Nat. Nanotechnol. **9**, 780 (2014).
